# Knowledge distillation-based rapid identification of Ligustici Rhizoma et Radix decoction piece varieties

**DOI:** 10.3389/fphar.2026.1923842

**Published:** 2026-10-22

**Authors:** Haoyu Hu, Shangzhou Jiang, Shengting Liang, Wentao Zhong, Shenglin Luo, Sijie Liu, Ruifen Hao, Geng Li

**Affiliations:** 1 State Key Laboratory for Quality Ensurance and Sustainable Use of Dao-Di Herbs, Institute of Chinese Materia Medica, China Academy of Chinese Medical Sciences, Beijing, China; 2 School of Pharmacy, Jiangxi University of Chinese Medicine, Nanchang, Jiangxi, China; 3 Modern Research Center for Traditional Chinese Medicine, Shanxi University, Taiyuan, Shanxi, China

**Keywords:** artificial intelligence, Automation, computer programming, convolutional neural network, traditional Chinese medicine decoction pieces

## Abstract

To address the commingling of different varieties of *Ligusticum* decoction pieces in the market and the difficulty of achieving rapid, efficient, and accurate on-site identification, this study aimed to develop a lightweight deep learning model that combines high identification accuracy with fast inference speed. Methods: Images of six categories of *Ligusticum* decoction pieces and their adulterants were acquired in an enclosed photographic light box to construct a standardized image dataset. Using ImageNet-pretrained weights, several high-accuracy models were selected as teacher models through comparative model evaluation, and knowledge distillation was subsequently employed to optimize the lightweight MobileNetV2 model as the student model. Finally, an interactive identification application was developed using PyQt5. Results: After knowledge distillation, the MobileNetV2 model achieved an identification accuracy of 99.73%, approaching the accuracy of the teacher models. Meanwhile, the distilled model contained only 2.23 million parameters, substantially fewer than the teacher models, thereby achieving both high identification accuracy and ease of deployment. Conclusion: The lightweight identification model developed in this study efficiently extracted high-level discriminative features from images of *Ligusticum* decoction pieces and their adulterants, enabling rapid and accurate identification and providing a feasible technical approach for the rapid on-site authentication of traditional Chinese medicine decoction pieces.

## 1 Introduction

*Ligustici Rhizoma et Radix* is the dried rhizome and root of *Ligusticum sinense* Oliv. or *Ligusticum jeholense* Nakai et Kitag., both belonging to the family Apiaceae. It was first documented in *Shennong’s Classic of Materia Medica* and classified as a middle-grade medicinal substance (Chinese Pharmacopoeia Commission, 2025). Characterized by a pungent taste and warm nature, it enters the Bladder meridian and is traditionally used to dispel wind, disperse cold, eliminate dampness, and relieve pain. Clinically, it is commonly used for the treatment of wind-cold common cold, vertex headache, and wind-dampness arthralgia, and is regarded as an important medicinal material for the treatment of vertex headache (National Administration of Traditional Chinese Medicine and Editorial Committee of Chinese Materia Medica, 1999).

In recent years, the increasing market demand for *Ligustici Rhizoma et Radix* has led to the gradual depletion of its wild resources, while cultivated materials have progressively become predominant in the market. However, the current quality standards for *Ligustici Rhizoma et Radix* do not adequately reflect the macroscopic characteristics of the cultivated materials commonly available in the market, making it difficult to effectively identify and distinguish them from major adulterants. Moreover, descriptions of the morphological characteristics of *Ligustici Rhizoma et Radix* in ancient materia medica literature are relatively brief, and the morphological similarities among materials of multiple botanical origins and closely related species have resulted in long-standing confusion regarding their identification and use (Zheng et al., 2024; Ding, 2006; Sun et al., 2018; Qiao et al., 2022). Although various techniques are currently available for the authentication of *Ligustici Rhizoma et Radix*, they still have notable limitations. Traditional macroscopic and microscopic identification methods are highly subjective and often exhibit limited reproducibility, whereas modern instrumental methods, although scientifically rigorous and objective, generally require poorly portable equipment and relatively long analytical procedures. Neither technique can fulfill the on-site identification demands in circulation scenarios. Consequently, *Ligustici Rhizoma et Radix* lacks efficient control approaches for frontline market circulation and quality supervision, which further aggravates the coexistence of mixed varieties and uneven quality of medicinal materials. It is clear that the core conflict in the identification of *Ligustici Rhizoma et Radix* varieties is not the scarcity of identification methods, but the failure of existing technologies to meet core requirements such as rapidity, precision and objectivity under various scenarios. Hence, it is imperative to capitalize on the simplicity and high efficiency of traditional identification, overcome its limitations with modern technologies, and construct an on-site rapid and precise identification system for Chinese medicinal materials and processed decoction pieces containing adulterants (Zheng et al., 2024; Zheng et al., 2024; Li et al., 2024).

The genus *Ligusticum* L. (Apiaceae) comprises approximately 60 species worldwide, of which roughly 30 occur in China. Its generic delimitation has long been problematic: molecular phylogenetic evidence based on nuclear ribosomal and plastome sequences has demonstrated that the genus is rampantly polyphyletic, with Chinese species dispersed across multiple distantly related clades (Zhou et al., 2020; Ren et al., 2022). The circumscription of *Ligusticum* sensu stricto has consequently been drastically reduced, while most Chinese species await formal revision (Zhou et al., 2020; Ren et al., 2025) - a nomenclatural instability that directly complicates the authentication of medicinal materials.

Two groups are directly relevant to this study. First, the taxa traded as “Xinjiang Gaoben” and “Huang Gaoben” have been transferred to Conioselinum (C. vaginatum and C. tataricum), yet remain morphologically similar to *Ligusticum* decoction pieces owing to the close affinity of both genera within tribe Selineae (Pimenov et al., 2001; Zhou et al., 2020). Second, L. chuanxiong Hort., known exclusively from cultivation with no confirmed wild progenitor, is formally included under *Ligusticum* in the Pharmacopoeia of the Peoples Republic of China (2025 edition) (Chinese Pharmacopoeia Commission, 2025). Palynological evidence supports treating L. chuanxiong and closely related cultivated forms as derivatives of L. sinense (Wang, 1990)^,^ and morphological comparisons show regional L. chuanxiong closely resembles L. sinense in plant form, rhizome shape, and crude drug characters (Ma, 2009). Consequently, the decoction pieces of L. sinense, L. chuanxiong, and cv. Jinxiong are macroscopically difficult to distinguish, forming the core fine-grained discrimination challenge of this study.

Given these unresolved systematic issues, we adopt an operational taxonomic framework defining six operational taxonomic units (OTUs) - L. sinense, L. jeholense, L. chuanxiong, C. vaginatum, C. tataricum, and L. sinense cv. Jinxiong - each verified by dual authentication (expert morphological identification and ITS2 DNA barcoding), providing a stable classification basis independent of ongoing nomenclatural disputes.

Macroscopic external morphological traits serve as the most intuitive and readily available source of identification information for medicinal raw materials, featuring low sampling costs, freedom from elaborate preprocessing, and suitability for on-site inspection. Combining these visual morphological features with deep learning algorithms, with digital images as input and species classification results as output, constitutes the optimal technical strategy to realize rapid, objective and precise variety discrimination of medicinal materials.

Convolutional Neural Networks (CNNs) were first established with the proposal of LeNet-5 by LeCun et al., in 1998, which laid the fundamental architecture of CNNs. In 2012, AlexNet triggered the vigorous development of CNNs by adopting ReLU activation functions, Dropout regularization and GPU parallel training (Krizhevsky et al., 2017), marking a landmark breakthrough for this field. Afterwards, a host of high-performance backbone networks including GoogLeNet, VGG and ResNet were proposed one after another (Simonyan and Zisserman, 2015; He et al., 2016). Benefiting from the rapid advancement of computer hardware, deep learning-based object detection technologies have achieved remarkable progress. Many researchers have applied such algorithms to the varietal identification of traditional Chinese medicinal materials and obtained phased research achievements, which preliminarily verified the feasibility of deep learning for medicinal material image classification (Zheng et al., 2024; Li et al., 2024; Chen et al., 2023). Nevertheless, the backbone networks adopted in the existing studies are generally computation-intensive and parameter-intensive. Despite their high classification accuracy, these models heavily rely on GPU computing resources and cannot be directly deployed on portable or embedded devices with limited hardware resources. In contrast, lightweight models such as the MobileNet series can deliver efficient on-device inference, yet they suffer from limited model capacity and inadequate feature extraction capability (Howard et al., 2017; Sandler et al., 2018; Si and Qi, 2023; Huang et al., 2022). Leading to unsatisfactory accuracy for fine-grained classification tasks targeting *Ligustici Rhizoma et Radix* and its closely related adulterants. Accordingly, how to equip lightweight models with discrimination accuracy comparable to deep heavyweight networks without sacrificing deployment efficiency has become the core bottleneck restricting the practical application of deep learning in on-site identification of *Ligustici Rhizoma et Radix.*

The emergence of Knowledge Distillation technology offers a novel solution to address the aforementioned dilemma. Recently, (Hou et al., 2025) proposed ShuffleCANet, a knowledge distillation framework employing DenseNet and RegNet as dual teachers and a cross-attention-enhanced ShuffleNet as the student model, achieving 98.8% classification accuracy on a 20-class Chinese botanical drug medicine recognition task, thereby demonstrating the viability of multi-teacher distillation for botanical drug medicine image identification. Its core principle lies in transferring knowledge learned by high-precision, high-complexity deep networks (teacher models) into lightweight shallow networks (student models) via soft label supervision (Si and Qi, 2023; Huang et al., 2022; Hinton et al., 2015). This enables student models to inherit the powerful feature representation capability of teacher models while retaining their inherent strengths of small parameter size and fast inference speed. Compared with other lightweight strategies including model compression, quantization and pruning, Knowledge Distillation stands out as it preserves the original student model architecture without sacrificing inference efficiency; it merely boosts model accuracy through teacher supervision signals during training, making it particularly suitable for fine-grained image recognition tasks under resource-limited conditions.

Based on the above analysis, this study takes the processed decoction piece images of *Ligustici Rhizoma et Radix* and its common adulterants as the dataset. We adopt VGG16, DenseNet169 and ResNet50 as teacher models and lightweight MobileNetV2 as the student model to construct a multi-teacher knowledge distillation framework. This work aims to realize rapid, accurate and portable on-site identification of *Ligustici Rhizoma et Radix* decoction pieces, and provide technical support for intelligent quality control during the circulation of traditional Chinese medicinal materials. (Figure 1).

**FIGURE 1:**
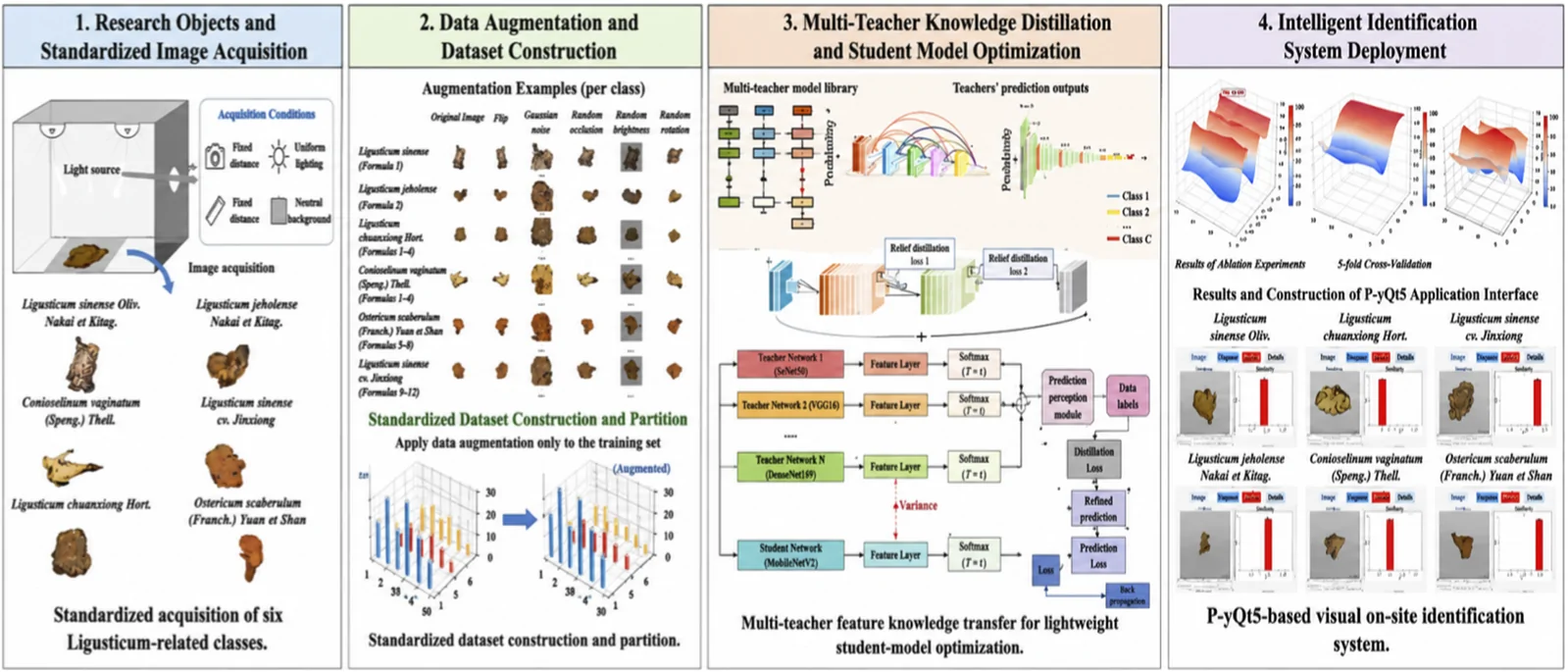
Overview of the proposed multi-teacher knowledge distillation framework for identifying *Ligusticum* decoction pieces and their adulterants.

## 2 Materials and methods

### 2.1 Dataset collection

The comprehensiveness and representativeness of the samples are critical to the reliability of the study. To minimize the potential effects of collection year and geographic origin, a multi-year and multi-regional sampling strategy was adopted. Over a 5-year period, a total of 120 batches representing six categories of *Ligustici Rhizoma et Radix* and its adulterants were collected from six provinces across China, including Zhonghua Gaoben, Liao Gaoben, Huang Gaoben, Xinjiang Gaoben, Chuanxiong, and Jinxiong. The botanical origins of the samples were authenticated through a combination of expert identification and DNA-based molecular identification to ensure the reliability of species determination. The nomenclature of the original plant species followed the 2025 edition of the *Chinese Pharmacopoeia*. The sample collection procedure is illustrated in Figure 2.

**FIGURE 2:**
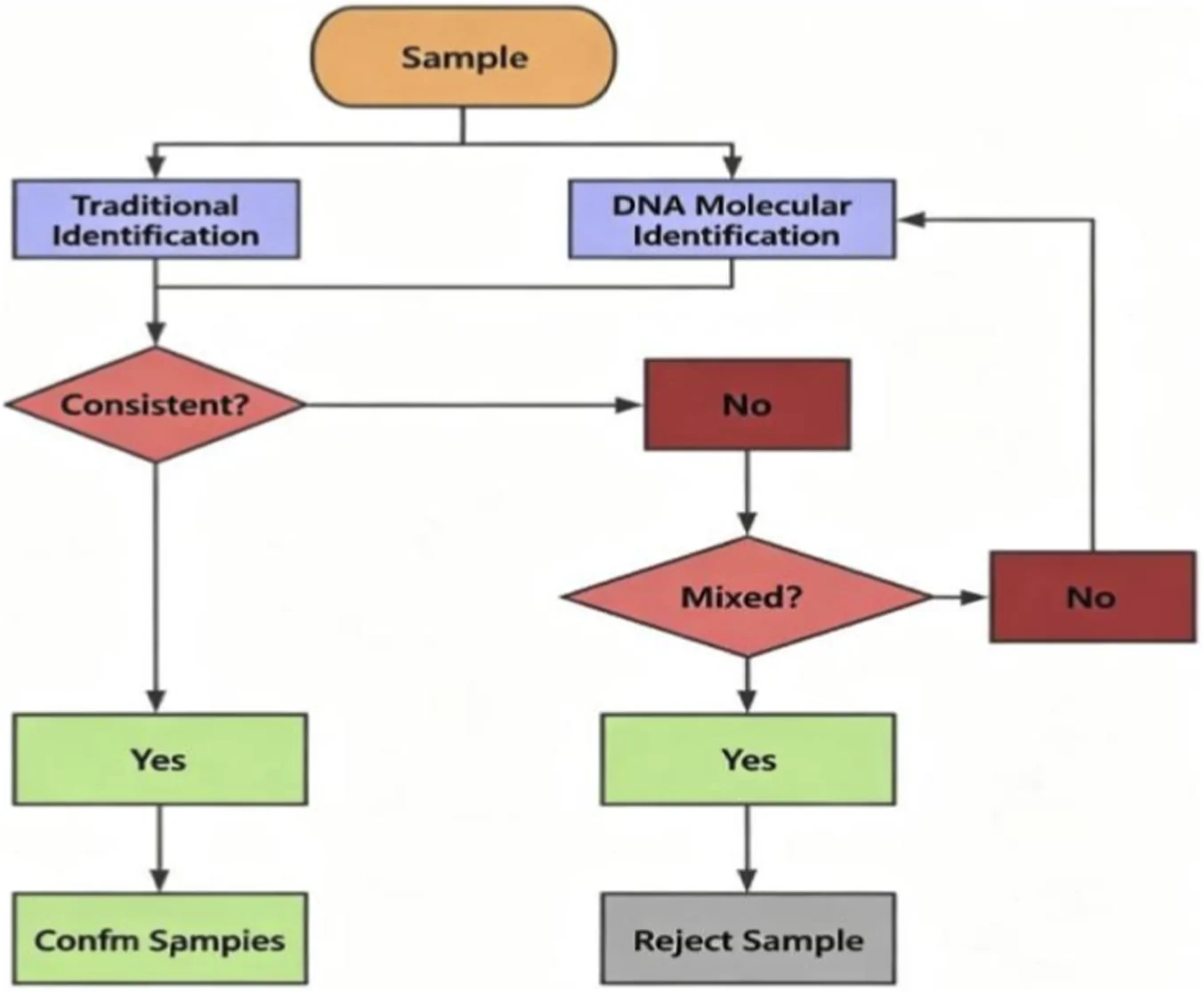
Sample collection procedure.

To minimize interference from weather conditions and ambient lighting, all images were acquired in an enclosed photographic light box under constant overhead illumination. The image acquisition system comprises a Light Hunter 800 image sensor (50 MP), a 320 × 320 mm textured light tent, a 1700 mm adjustable photographic bracket (Jiahuacai), and a constant-color-temperature LED fill light (120 × 75 mm, 12 W, 4000 K). A smartphone holder was used to maintain a fixed shooting distance and angle, and all images were captured at 2× zoom. The overall image acquisition setup is illustrated in Figure 3. A total of 7,363 images were collected from 120 batches of Ligustici Rhizoma et Radix pieces and their adulterants. The original image resolution is 3072 × 4096 (12.58 megapixels). The entire dataset was randomly split into training, validation, and test sets according to a ratio of 60%, 20%, and 20%, respectively. The detailed dataset distribution is shown in Table 1.

**FIGURE 3:**
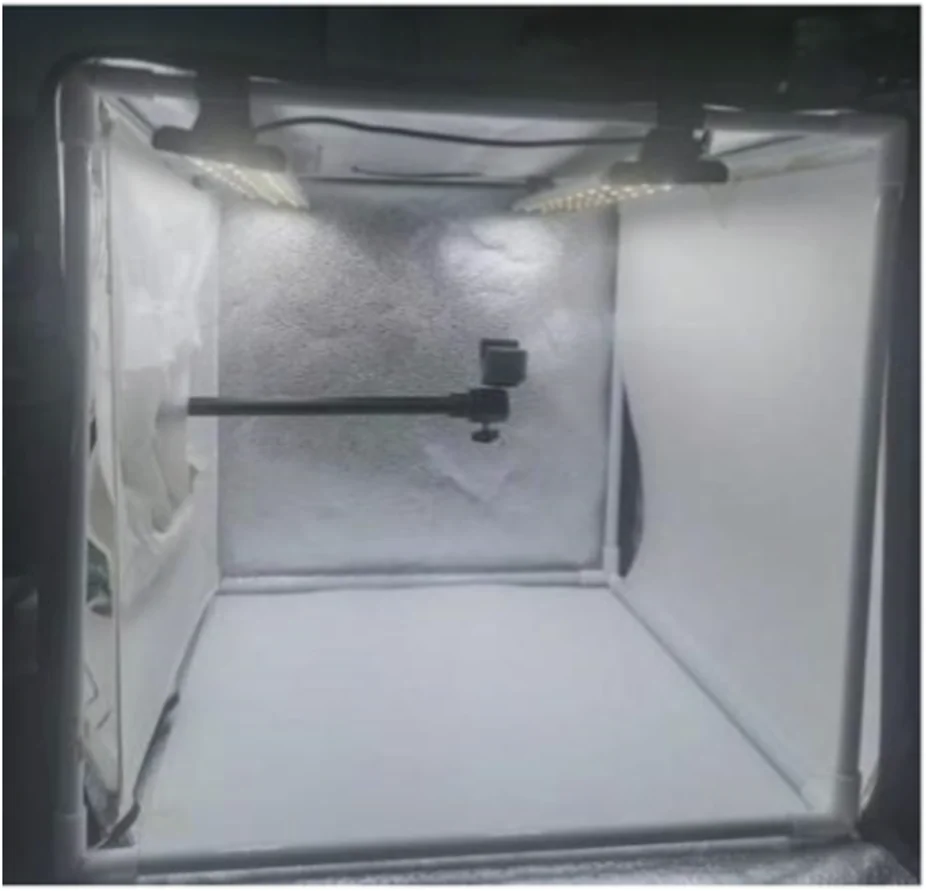
Image acquisition device for *Ligusticum sinense* decoction pieces.

**TABLE 1:** Summary of the original dataset.

Class		Variety	Train set	Val set	Test set	Total
Decoction pieces	Authentic products	*Ligusticum sinense*Oliv	667	223	222	1112
		*Ligusticum jeholense*Nakai et Kitag	1009	337	337	1683
	Confusable adulterants	*Ligusticum chuanxiong* Hort	664	222	222	1108
	Adulterants	*Conioselinum vaginatum* (Spreng.) Thell	1027	343	342	1712
		*Ostericum scaberulum* (Franch.) Yuan et Shan	705	236	235	1176
		*Ligusticum sinense* cv. Jinxiong	343	115	114	572
Total		6 classes	4415	1476	1472	7363

The model training and inference experiments were conducted on a workstation equipped with an Intel Core i9-14900 KF CPU and an NVIDIA GeForce RTX 5080 GPU (16 GB VRAM), running Windows 11 as the operating system. The software environment required for deep learning and interface development includes: Windows 10; Python 3.10; CUDA 12.9; PyTorch 2.3.1 + cu121; PyQt5; Matplotlib; and cuDNN 8.9.7. The photographic setup consists of a lychee-pattern photographic reflector box (320 × 320 mm), a Jiahua Cai adjustable photographic tripod, and constant color temperature LED lights; the detailed configuration is presented in Table 2.

**TABLE 2:** List of instruments and reagents.

Equipment name	Brand	Model and parameters
Smartphone camera	Redmi	K70, equipped with light Hunter 800 sensor
Lychee-texture photographic light box	Selens	320 × 320 mm
Adjustable photography tripod	Jiahua cai	1700 mm
Constant-temperature LED fill light	Jingyuguang	120 × 75 mm
Central processing unit (CPU)	Intel	Core i9-14900KF, 24 cores & 32 threads
Graphics processing unit (GPU)	NVIDIA	GeForce RTX 5080, 16 GB VRAM

### 2.2 Image data preprocessing

The images were standardized to obtain a distribution with a mean of 0 and a standard deviation of 1. This procedure reduced scale-related differences, minimized the influence of data bias on learning efficiency, mitigated interference caused by affine transformations, and accelerated model convergence. For the three RGB channels, predefined mean values of [0.485, 0.456, 0.406] and standard deviations of [0.229, 0.224, 0.225], corresponding to the R, G, and B channels, respectively, were used. Mean subtraction and variance scaling were applied to all images. The preprocessing results are shown in Figure 4, and the standardization was calculated as follows (Equations 1–3):

$$I^{\prime }\left(I_{R}^{\prime },I_{G}^{\prime },I_{B}^{\prime }\right)=\left(I_{R}-\frac{\sum_{i}^{N}I_{i,R}}{N},I_{G}-\frac{\sum_{i}^{N}I_{i,G}}{N},I_{B}‐\frac{\sum_{i}^{N}I_{i,B}}{N}\right)$$ (1)

$$\left(\sigma_{R},\sigma_{G},\sigma_{B}\right)=\left(\frac{\sum_{i}^{N}{\left(I_{i,R}‐\frac{\sum_{i}^{N}I_{i,R}}{N}\right)}^{2}}{N},\frac{\sum_{i}^{N}{\left(I_{i,G}‐\frac{\sum_{i}^{N}I_{i,G}}{N}\right)}^{2}}{N},\frac{\sum_{i}^{N}{\left(I_{i,B}‐\frac{\sum_{i}^{N}I_{i,B}}{N}\right)}^{2}}{N}\right)$$ (2)

$$I^{\prime \prime }\left(I_{R}^{\prime \prime },I_{G}^{\prime \prime },I_{B}^{\prime \prime }\right)=\left(\frac{I_{R}^{\prime }}{\sigma_{R}},\frac{I_{G}^{\prime }}{\sigma_{G}},\frac{I_{B}^{\prime }}{\sigma_{B}}\right)$$ (3)

**FIGURE 4:**
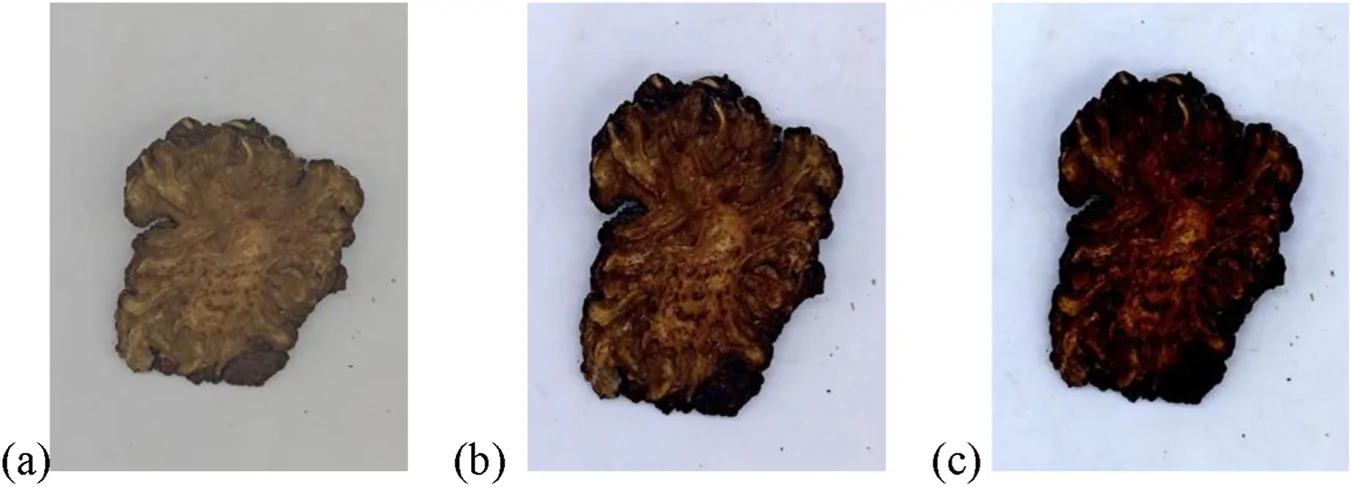
Illustration of the standard normalization results. **(a)** Original image **(b)** mean subtraction **(c)** normalization.

### 2.3 Data augmentation

Data quality and sample diversity are critical factors affecting the performance of deep learning models. To improve the generalization ability of the model for images of *Ligustici Rhizoma et Radix* and its adulterants and to reduce the risk of overfitting, data augmentation was applied to the training and validation sets. The augmentation methods included mirror flipping, 1.5-fold horizontal scaling, random rotation (±10°–180°), Gaussian blurring with a radius of 5, and random brightness adjustment within ±50%. (See Figure 5). The test set consisted exclusively of the original images without data augmentation to ensure an objective evaluation of model performance. The numbers of images obtained after applying the five augmentation methods are presented in Table 3.

**FIGURE 5:**
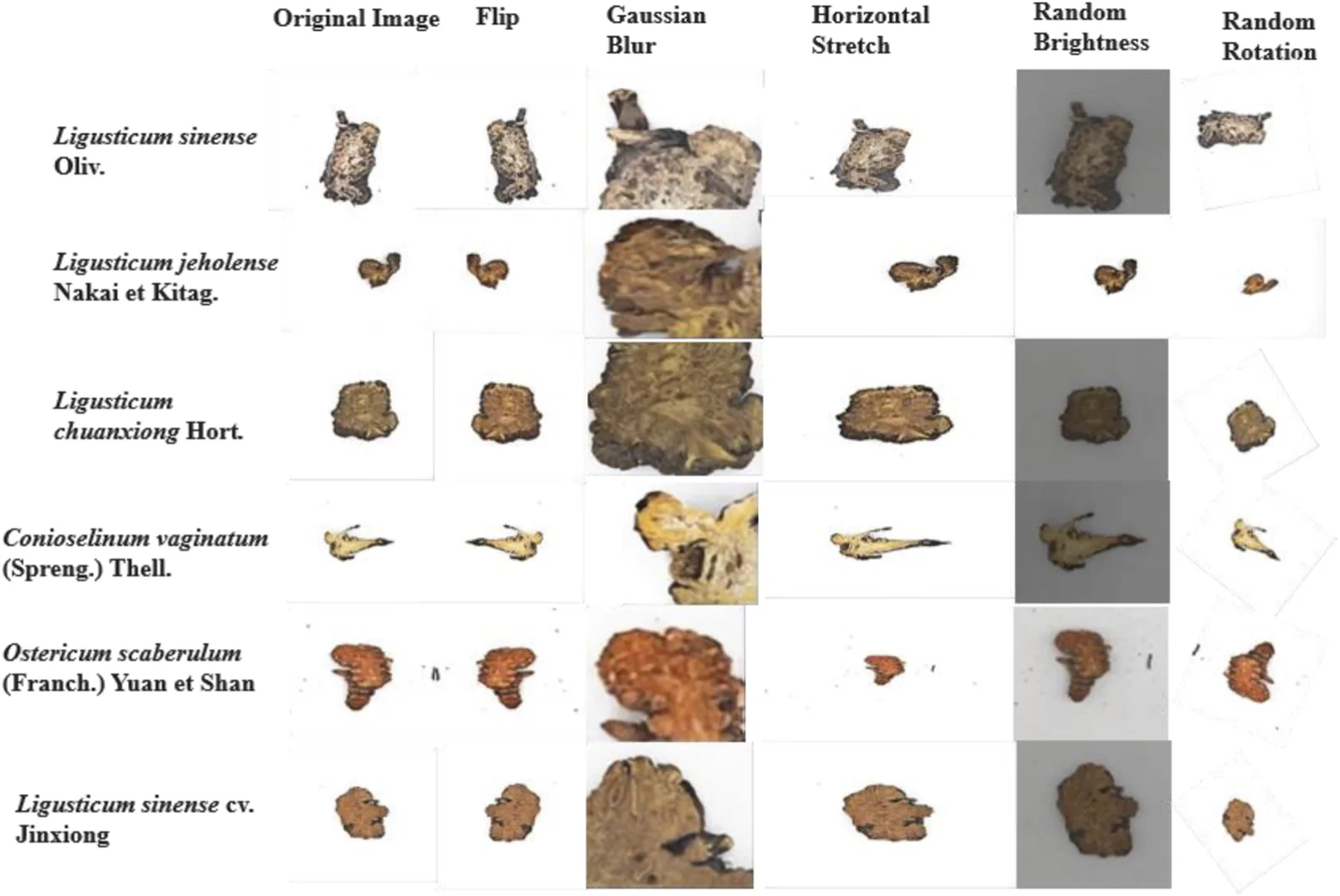
Data augmentation.

**TABLE 3:** Image augmentation of Ligustici Rhizoma et Radix and its adulterants.

Class		Variety	Train set	Val set	Test set	Total
Decoction pieces	Authentic products	*Ligusticum sinense*Oliv	4002	1338	222	5562
		*Ligusticum jeholense*Nakai et Kitag	6054	2022	337	8413
	Confusable adulterants	*Ligusticum chuanxiong* Hort	3984	1332	222	5538
	Adulterants	*Conioselinum vaginatum* (Spreng.) Thell	6162	2058	342	8562
		*Ostericum scaberulum* (Franch.) Yuan et Shan	4230	1416	235	5881
		*Ligusticum sinense* cv. Jinxiong	2058	690	114	2862
Total		6 classes	26,490	8856	1472	36,818

## 3 Research methods

### 3.1 Knowledge distillation

Neural network compression methods can generally be classified into four categories: network pruning, weight quantization, weight decomposition, and knowledge distillation (KD) (Si and Qi, 2023; Huang et al., 2022). Knowledge distillation adopts a teacher–student training framework, in which one or more pretrained teacher models provide knowledge that is transferred to a student model through distillation-based training (Huang et al., 2022; Hinton et al., 2015). This approach enables knowledge learned by a complex teacher model to be transferred to a simpler student model, typically at the cost of only a minor reduction in performance.

Knowledge distillation was first proposed by Hinton et al., in 2015 and can be broadly divided into single-teacher and multi-teacher knowledge distillation (Hinton et al., 2015). In 2017, You et al. introduced a multi-teacher knowledge distillation strategy in which multiple teacher models jointly guided the student model by averaging their outputs (National Administration of Traditional Chinese Medicine and Editorial Committee of Chinese Materia Medica, 1999). By integrating the knowledge and capabilities of multiple teacher models, multi-teacher knowledge distillation can improve the generalization ability and robustness of the student model compared with single-teacher distillation.

For the identification of *Ligustici Rhizoma et Radix*, both high identification accuracy under laboratory conditions and efficient deployment in edge-device scenarios, such as medicinal-material procurement and retail pharmacies, need to be considered. Such application scenarios are generally constrained by limited computational resources and therefore impose stringent requirements on model size and real-time inference efficiency. Meanwhile, the subtle morphological differences among *Ligustici Rhizoma et Radix* decoction pieces and their adulterants require the model to possess a strong feature extraction capability. A single model architecture is therefore unlikely to achieve an optimal balance between these competing requirements. Large-scale models generally achieve high identification accuracy but contain numerous parameters, making them difficult to deploy on edge devices. In contrast, lightweight models are more suitable for deployment because of their smaller parameter sizes, but their identification accuracy is often lower. Although both types of models have distinct advantages, neither can fully satisfy the requirements of all application scenarios (Howard et al., 2017; Sandler et al., 2018; Si and Qi, 2023; Huang et al., 2022).

To address this issue, a heterogeneous multi-teacher knowledge distillation framework was constructed in this study. The complementary advantages of multiple teacher models were transferred to a lightweight student model, with the aim of simultaneously achieving high identification accuracy and ease of deployment for the authentication of *Ligustici Rhizoma et Radix* (Table 4). The overall framework is illustrated in Figure 6.

**TABLE 4:** Parameter settings.

Parameters	Value or type
Random Seed	42
Initial learning rate	1e-4
Final learning rate	1e-5
LR Scheduler	CosineAnnealingLR (T_max = 30)
Epochs	50
Batch size	16
Weight-decay	1e-3
Baseline ModelDroupt	0.5
Teacher ModelDroupt	0.8
Student ModelDroupt	0.85
Optimizer	Adam
Distillation loss	Cross-entropy loss (hard loss) + KL Divergence loss (soft loss)
T/α	2.0/0.5

**FIGURE 6:**
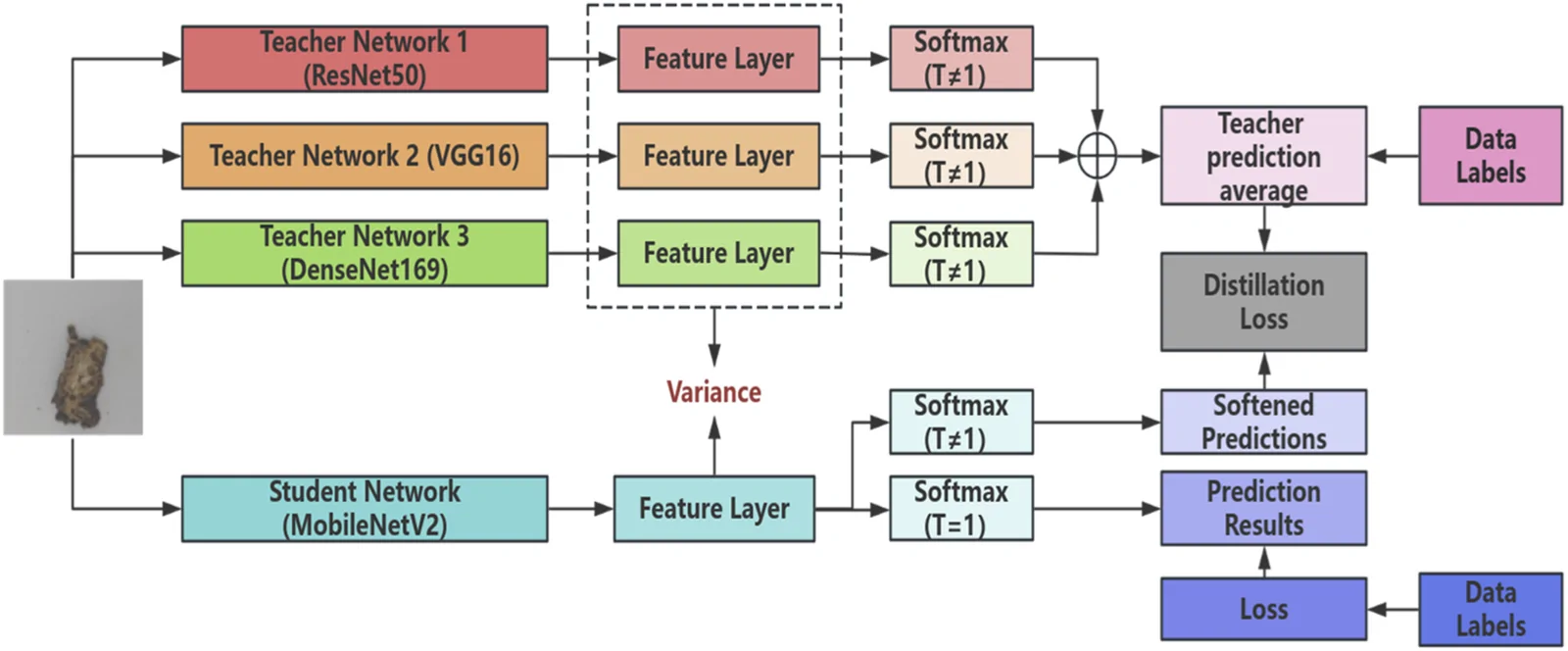
Multi-teacher knowledge distillation framework.

### 3.2 Teacher models

#### 3.2.1 ResNet50

ResNet, or residual network, is a deep convolutional neural network proposed by He et al., in 2015 (He et al., 2016). Among its variants, ResNet50 has become one of the most widely used architectures in the field of image recognition. The overall network consists of an input preprocessing layer, five main feature extraction stages containing residual blocks, a global average pooling layer, and a classification head.

The principal innovation of ResNet lies in its residual learning mechanism. ResNet architectures mainly employ two types of residual blocks: BasicBlock and Bottleneck. ResNet50 adopts the Bottleneck structure to reduce computational complexity and improve parameter efficiency. Each Bottleneck block consists of a sequence of 1 × 1, 3 × 3, and 1 × 1 convolutional layers. The first 1 × 1 convolution reduces the number of channels, thereby decreasing computational cost. The subsequent 3 × 3 convolution extracts spatial features, whereas the final 1 × 1 convolution restores the channel dimension, allowing the resulting feature map to be combined with the input feature map (X) through a residual connection. The structures of the BasicBlock and Bottleneck residual blocks are illustrated in Figure 7 (He et al., 2016).

**FIGURE 7:**
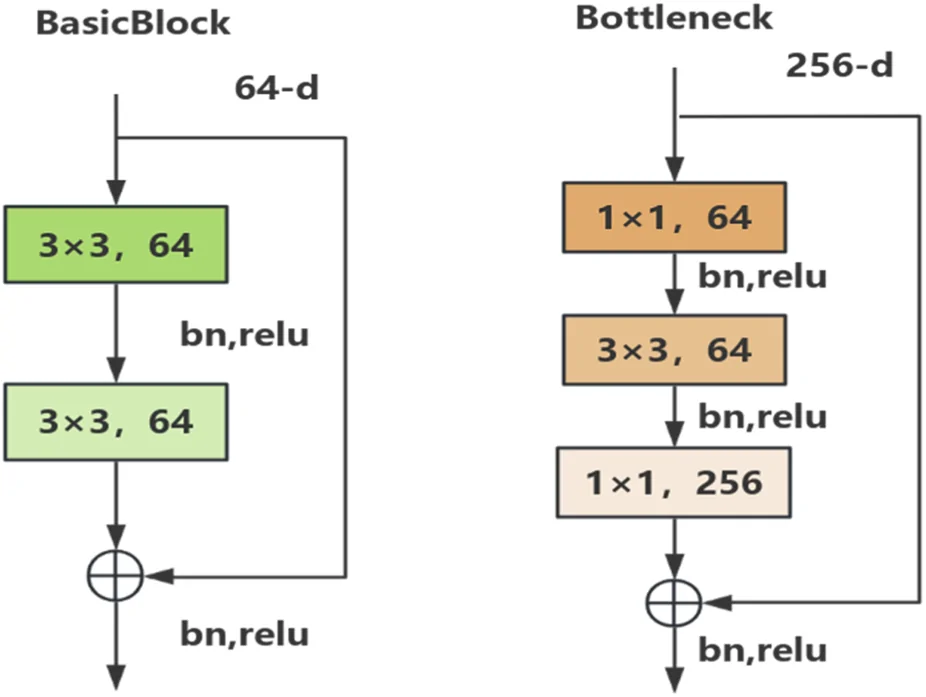
Residual block structures.

The residual mapping is expressed as follows Equation 4:

$$H\left(x\right)=F\left(x\right)+x$$ (4)

where (x) denotes the input feature map, (F(x)) represents the mapping obtained through convolution and activation operations, and (H(x)) denotes the output of the residual unit.

The residual connection (RC) preserves the feature extraction capability of the earlier network layers and alleviates the problems of vanishing and exploding gradients, thereby enabling substantial performance improvements in large-scale image recognition tasks such as ImageNet classification. The overall architecture of ResNet50 is illustrated in Figure 8.

**FIGURE 8:**
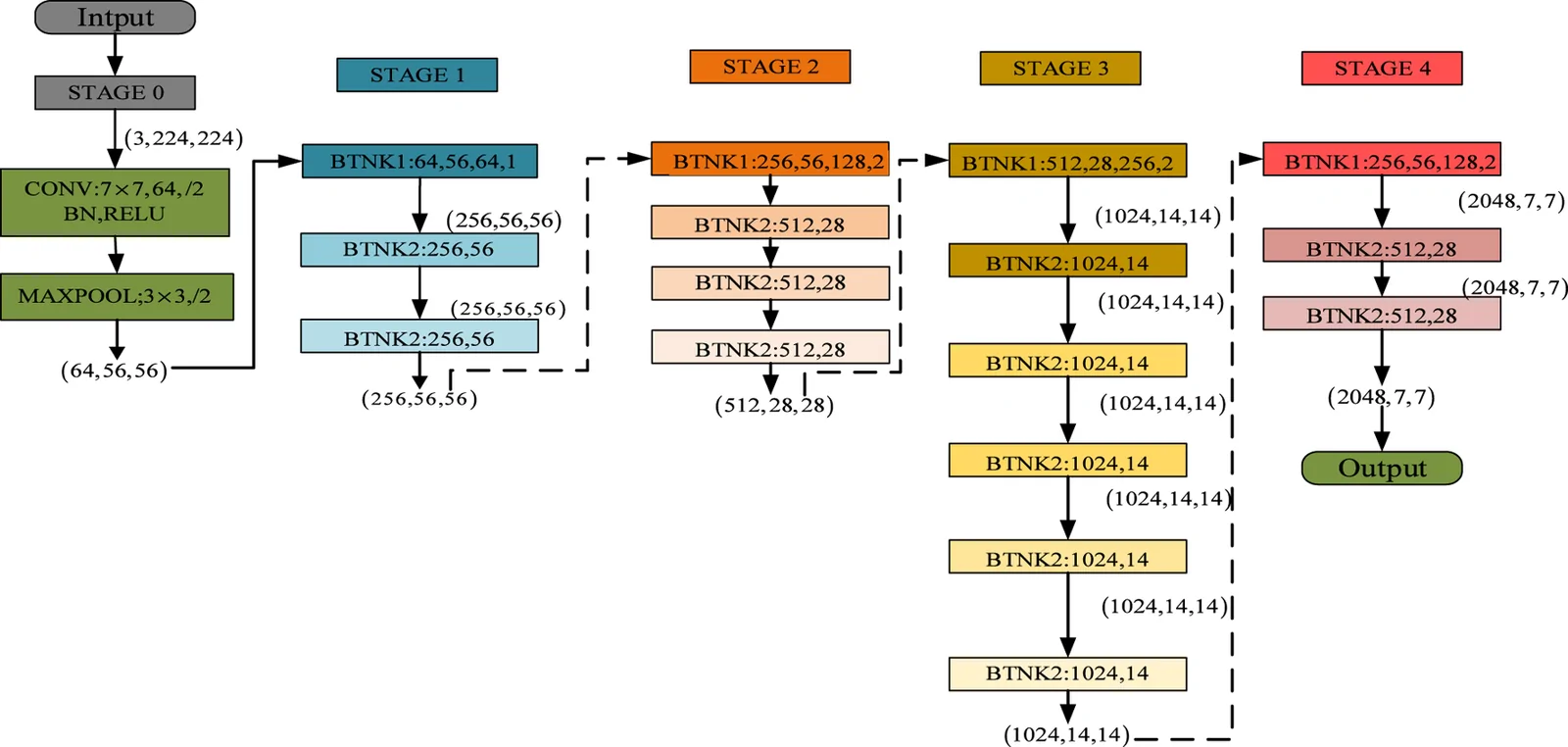
Architecture of ResNet50.

#### 3.2.2 DenseNet169

DenseNet, or densely connected convolutional network, is a deep convolutional neural network proposed by Huang et al., in 2017. By introducing a dense connectivity mechanism, DenseNet effectively alleviates the vanishing-gradient problem and enhances the feature extraction capability of the network. DenseNet169 is a representative architecture in the DenseNet family that achieves a favorable balance between classification accuracy and computational efficiency. It has been widely applied to computer vision tasks, including image classification and feature extraction. The overall architecture of DenseNet169 comprises five main components: an input preprocessing layer, dense blocks, transition layers, a global average pooling layer, and a classification head (Zhang and Wang, 2025).

The Dense Block is the core component of DenseNet. Each Dense Block consists of multiple dense layers that are connected in a densely connected manner. The output feature maps of each layer are used as inputs to all subsequent layers, thereby facilitating feature reuse and cross-layer information fusion. This dense connectivity mechanism improves information and gradient propagation throughout the network, contributes to stable model training, and enhances classification performance. A schematic representation of the Dense Block is shown in Figure 9 (Zhang and Wang, 2025).

**FIGURE 9:**
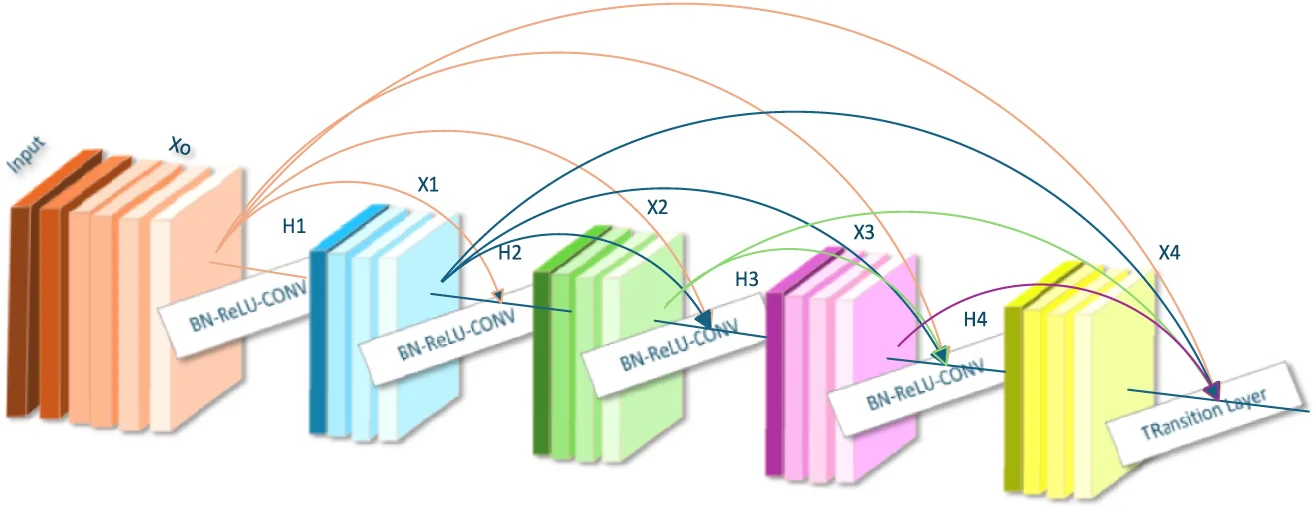
Dense block of DenseNet169.

The dense connectivity mechanism can be expressed as follows (Equation 5):

$$\text{Xl}=\text{Hl}\left(\left[X0,X1,X2,...,\text{Xl}-1\right]\right)$$ (5)

where (Xl) denotes the output feature map of the Hl represents the feature transformation function containing the Bottleneck structure; and ([X0,X1,X2,Xl-1]) denotes the concatenation of the initial input (X0) and the output feature maps of all preceding layers, including (X1) from the first layer and (X2) from the second layer.

#### 3.2.3 VGG16

VGG is a convolutional neural network architecture proposed by Simonyan and Zisserman (Simonyan and Zisserman, 2015). By stacking multiple convolutional layers with small 3 × 3 kernels and employing regular downsampling operations, the network increases its depth and progressively extracts high-level image features. This architecture enhances deep feature representation and can help reduce the risk of overfitting when appropriate regularization strategies are applied.

VGG16 is one of the most representative architectures in the VGG family. Its overall network consists of an input preprocessing layer, five convolutional and pooling blocks, three fully connected layers, and a classification output layer. The architecture of VGG16 is illustrated in Figure 10.

**FIGURE 10:**
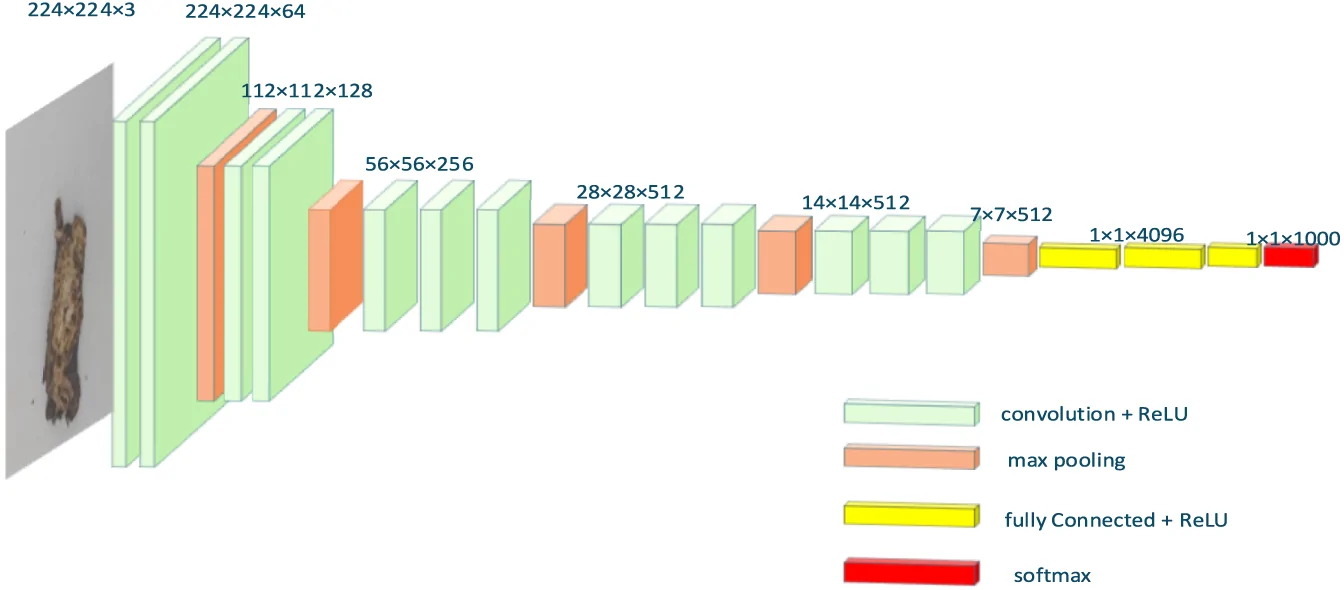
Convolutional and pooling block of VGG16.

The core architecture of VGG is based on modular convolutional blocks constructed by stacking small 3 × 3 convolutional kernels, followed by regularly arranged 2 × 2 max-pooling layers and fully connected layers. This design ensures effective feature extraction while establishing a simple and highly standardized network architecture (Simonyan and Zisserman, 2015).

### 3.3 Student model

#### 3.3.1 MobileNetV2

MobileNet was proposed by Andrew G. Howard et al. at Google. As the second-generation architecture of the MobileNet family, MobileNetV2 incorporates linear bottlenecks, inverted residual blocks, and optimized depthwise separable convolutions to reduce feature information loss and computational cost in conventional lightweight networks. Its overall architecture consists of four main components: an initial convolutional layer, a sequence of inverted residual bottleneck blocks, a global average pooling layer, and a classification output layer (Howard et al., 2017; Sandler et al., 2018).

The inverted residual block substantially reduces the computational cost and number of model parameters while maintaining effective feature representation. It consists of a 1 × 1 expansion convolution, a 3 × 3 depthwise convolution, and a 1 × 1 projection convolution. Its key components are the linear bottleneck, depthwise separable convolution, and shortcut connection. A schematic representation of the inverted residual block is shown in Figure 11 (Sandler et al., 2018).

**FIGURE 11:**
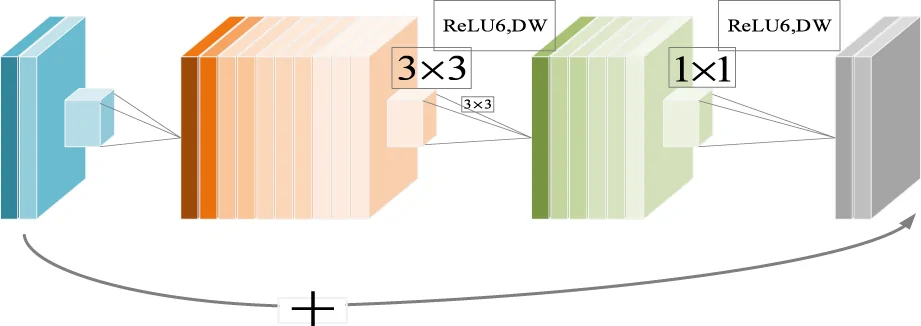
Inverted residual block.

The corresponding calculation is expressed as follows Equation 6:

$$\left(y=\left\{\begin{array}{ll} x+Linear\left({DWConv}_{3\times 3}\left({Conv}_{1\times 1}^{t}\left(x\right)\right)\right) & if\;s=1,Cin=Cout\\ Linear\left({DWConv}_{3\times 3}\left({Conv}_{1\times 1}^{t}\left(x\right)\right)\right) & if\;s=2\;or\;Cin=Cout \end{array}\right.\right)$$ (6)

where (y) denotes the output feature map of the inverted residual block; (x) denotes the input feature map; ${Conv}_{1\times 1}^{t}$ :1 × 1 *expansion convolution with an expansion factor of (t);* ${DWConv}_{3\times 3}$ :3 × 3 depthwise convolution; Linear :represents the linear projection convolution; (s) denotes the stride; and C_in_/C_out_:and denote the numbers of input and output channels, respectively.

### 3.4 Multi-teacher fusion distillation mechanism

A multi-teacher–student framework was adopted for knowledge distillation. Its central principle is to jointly train the student model using soft labels generated from the output probability distributions of multiple teacher models and hard labels derived from the ground-truth class annotations. Through this strategy, the student model can acquire the collective discriminative knowledge of multiple teacher models while remaining aligned with the true class labels, thereby satisfying both the high-accuracy identification requirements for *Ligustici Rhizoma et Radix* and its adulterants and the lightweight deployment requirements of different edge-device scenarios (Hinton et al., 2015; Hou et al., 2025).

#### 3.4.1 Dual-loss fusion distillation mechanism

To improve the efficiency of knowledge transfer in multi-teacher knowledge distillation, a dual-loss fusion mechanism was employed. The hard-label loss was used to maintain the fundamental classification performance, whereas the soft-label loss was used to transfer the generalized knowledge of the multiple teacher models. The two losses were subsequently combined using a weighted strategy, enabling the student model, MobileNetV2, to efficiently learn knowledge from the teacher models, namely, ResNet50, VGG16, and DenseNet169.

1. Distillation loss: Kullback–Leibler (KL) divergence was adopted as the distillation loss. Its primary purpose was to enable the student model to learn the generalized knowledge encoded by multiple teacher models rather than relying solely on the ground-truth labels. First, a temperature parameter (T) was introduced to soften the output probability distributions of the teacher models, thereby revealing the relative relationships among different classes and enriching the knowledge conveyed by the output distributions. Subsequently, the soft labels generated by the three heterogeneous teacher models were fused using a weighted strategy. This approach integrated the deep semantic representation capability of ResNet50, the local feature extraction capability of VGG16, and the feature reuse advantage of DenseNet169 to generate more comprehensive soft-label supervision. The soft-label loss was calculated using Equation 1; (Si and Qi, 2023; Huang et al., 2022; Hinton et al., 2015) (Equation 7):

$$\left(L_{soft}=KL\left(LogSoftmax\left(\frac{z_{s}}{T}\right)/bigg\|\sum_{i=1}^{3}w_{i}\cdot Softmax\left(\frac{z_{t_{i}}}{T}\right)\right)\cdot T^{2}/tag1\right)$$ (7)

2. Student loss: The conventional cross-entropy loss was adopted as the student loss to constrain the consistency between the student model predictions and the ground-truth labels. This loss preserves the fundamental classification capability of the student model and prevents it from excessively fitting the potentially biased knowledge transferred by the teacher models. The hard-label loss was calculated using (Equation 8):

$$\left(L_{hard}=‐\frac{1}{N}\sum_{n=1}^{N}\sum_{c=1}^{C}y_{n,c}\cdot \log\left(p_{n,c}\right)/tag2\right)$$ (8)

3. Weighted fusion loss: The soft-label loss and hard-label loss were combined using a weighting coefficient to construct the final knowledge distillation loss function. This weighted formulation balances the contributions of knowledge transfer from the teacher models and supervision from the ground-truth labels. The total loss was calculated as follows (Huang et al., 2022; Hinton et al., 2015; Hou et al., 2025) (Equation 9):

$$\left(L_{total}=\alpha \cdot L_{hard}+\left(1‐\alpha \right)\cdot L_{soft}/tag3\right)$$ (9)

## 4 Experimental details

### 4.1 Software environment

All model training, inference, and evaluation experiments were conducted on a workstation equipped with an Intel Core i9-14900 KF CPU and an NVIDIA GeForce RTX 5080 GPU with 16 GB VRAM, running the Windows 11 operating system. The experimental software environment includes Python 3.10, CUDA 12.9, cuDNN 8.9.7, and the PyTorch 2.3.1+cu121 deep learning framework. Among them, Matplotlib was used for data visualization, and PyQt5 was applied for interactive interface development. The entire processes of model training, inference and evaluation were implemented under this unified environment.

### 4.2 Hardware environment

The experimental platform was equipped with a 14th-generation Intel® Core™ i9-14900 KF processor, an NVIDIA GeForce RTX 5080 graphics processing unit, and 64 GB of DDR4-3600 memory configured as four 16 GB modules. This hardware configuration provided the computational resources required for model training and inference.

### 4.3 Experimental parameters

The loss functions and optimizers used in the experiments were selected according to the characteristics and training requirements of each model.

To address class imbalance in the dataset, weighted random sampling was implemented using the WeightedRandomSampler.Samples from minority classes were assigned higher sampling weights, whereas those from majority classes were assigned lower weights. This strategy increased the probability that samples from different classes contributed more evenly to gradient updates during model training, thereby reducing the tendency of the model to favor majority classes and lowering the risk of missed or incorrect classification of minority-class decoction pieces. It also contributed to more balanced overall classification performance. The class weights and sampling probabilities were calculated as follows Equations 11, 12:

$$\left(w_{i}=\frac{1}{n_{i}}\right)$$ (11)

$$\left(p_{j}=\frac{w_{l_{j}}}{\sum_{k=1}^{N}w_{l_{k}}}\right)$$ (12)

where (wi) denotes the sampling weight assigned to class (i); (ni) denotes the number of samples in class (i); (pj) denotes the sampling probability of sample (j); (lj) denotes the class label of sample (j); and (N) denotes the total number of samples.

### 4.4 Evaluation metrics

To evaluate the effectiveness of the distilled model, an evaluation framework was established from three perspectives: performance retention, compression efficiency, and inference efficiency. For performance retention, the loss value, accuracy (Acc), precision (Prec), recall (Rec), and F1-score were used to quantify classification performance. Compression efficiency was assessed based on the reductions in the number of model parameters (Params) and model size (Size). Inference efficiency was evaluated using inference time and the number of floating-point operations (FLOPs), thereby assessing the feasibility of deploying the distilled model on edge devices.

## 5 Experimental results

### 5.1 Comparison before and after knowledge distillation

This section presents the evaluation performance of the original MobileNetV2, ResNet50, DenseNet169, and VGG16 models, together with that of the distilled model, after 50 training epochs. The detailed performance of each model on the test set is presented in Table 5.

**TABLE 5:** Comparison between distilled model and baseline model.

Model	Performance evaluation				Model parameters		Inference efficiency	
	Acc	Prec	Rec	F1	M	Size	FPS	FLOPs(G)
MobileNetV2	89.33%	89.72%	88.54%	89.04%	2.23	8.75	312.72	0.33
DenseNet169	99.66	99.61	99.70	99.65	12.49	144	16.67	3.43
ResNet50	98.78%	98.44%	98.87%	98.64%	23.52	90.80	47.63	4.13
VGG16	99.18%	99.22%	99.15%	99.18%	134.29	512	238.37	15.47
Three-teacher knowledge distillation	99.73%	99.71%	99.70%	99.70%	2.23	8.75	294	0.33

The results showed that the three-teacher distilled model achieved the best performance across all evaluation metrics, with an accuracy of 99.73% and precision, recall, and F1-score values of no less than 99.70%. Its classification performance was substantially better than that of the other models. In terms of computational efficiency, the distilled model contained only 2.23 million parameters, occupied 8.75 MB of storage, required 0.33 GFLOPs per inference, and achieved an inference speed of 294 frames per second (FPS). These efficiency metrics were comparable to those of MobileNetV2, whereas its accuracy exceeded that of MobileNetV2 (89.33%) by more than 10 percentage points. Compared with DenseNet169, which achieved the highest accuracy among the teacher models at 99.66%, the distilled model maintained comparable accuracy while reducing the model size to 6.1% and the computational cost to 9.6% of those of DenseNet169. These results indicate that the three-teacher knowledge distillation strategy simultaneously maintained or slightly improved identification accuracy while achieving model compression and accelerated inference, thereby providing technical support for edge-device deployment and practical on-site identification of *Ligustici Rhizoma et Radix* decoction pieces and their adulterants.

### 5.2 Ablation experiments

To determine the contribution of each teacher model to the performance of the student model, seven comparative experiments were conducted using MobileNetV2 as the fixed student model. The distillation effects of the individual teacher models VGG16, DenseNet169, and ResNet50, different combinations of two teacher models, and the complete three-teacher combination were comparatively evaluated. The main evaluation dimensions included identification performance, as measured by accuracy (Acc), precision (Prec), recall (Rec), and F1-score; model lightweightness, as assessed by the number of parameters (M) and model storage size (Size); and inference efficiency, as evaluated by frames per second (FPS) and floating-point operations (FLOPs). The detailed experimental results are presented in Table 6, Figures 12, 13.

**TABLE 6:** Results of the ablation study.

Student model	Teacher model			Performance evaluation				Model parameters		Inference efficiency	
MobileNetV2	VGG16	DenseNet169	ResNet50	Acc(%)	Prec (%)	Rec (%)	F1 (%)	M	Size	FPS	FLOPs
√	√	​	​	96.20	96.54	95.79	96.10	2.23	8.75	298	0.33
√	​	√	​	97.21	97.39	97.14	97.25	2.23	8.75	286	0.33
√	​	​	√	99.32	99.06	99.35	99.20	2.23	8.75	326	0.33
√	√	√	​	95.92	95.65	96.33	95.87	2.23	8.75	294	0.33
√	√	​	√	98.85	98.78	98.82	98.80	2.23	8.75	308	0.33
√	​	√	√	98.78	98.19	98.76	98.45	2.23	8.75	291	0.33
√	√	√	√	99.73	99.71	99.70	99.70	2.23	8.75	294	0.33

**FIGURE 12:**
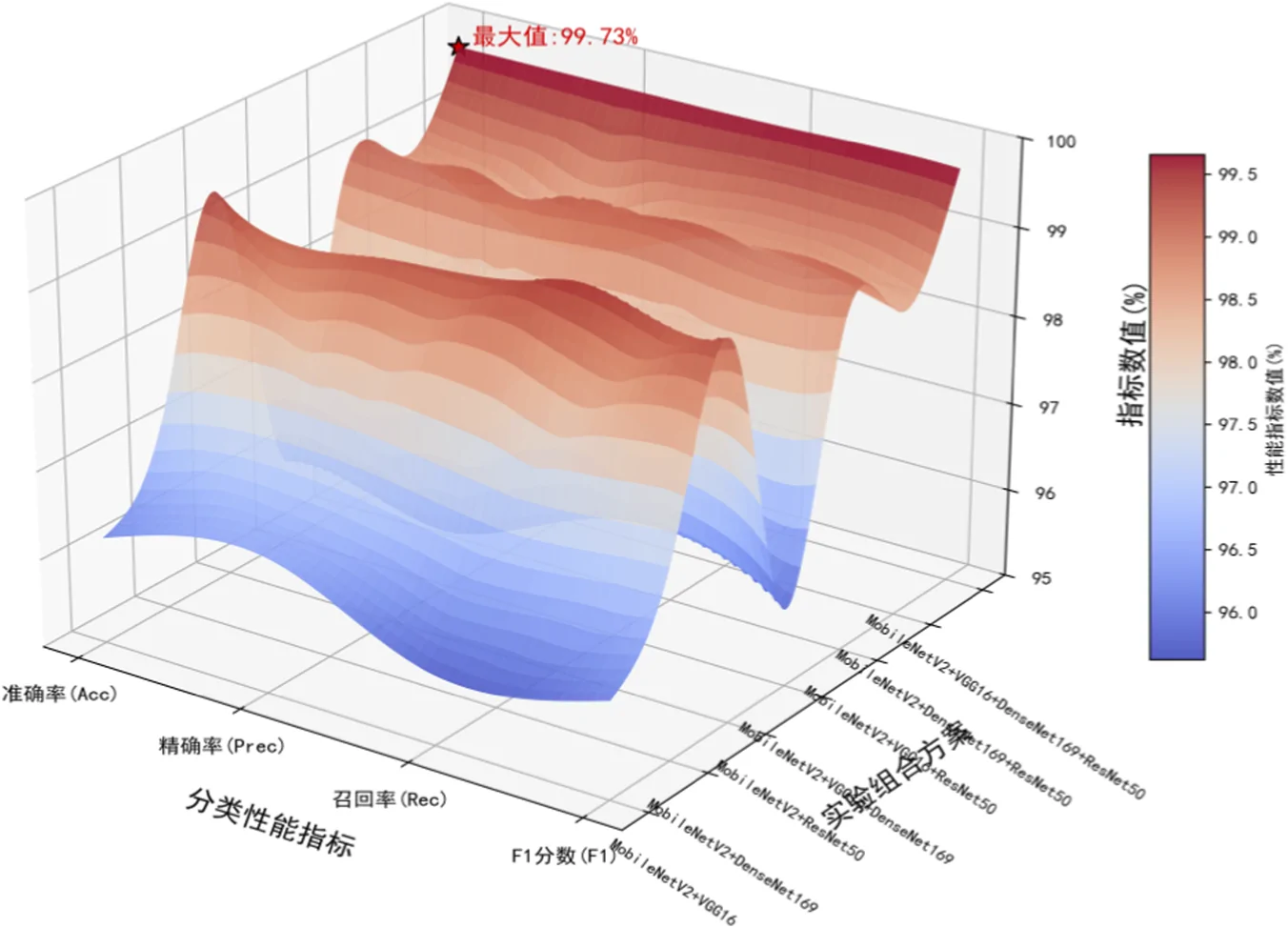
3D surface visualization results of ablation experiments on multi-teacher knowledge distillation schemes.

**FIGURE 13:**
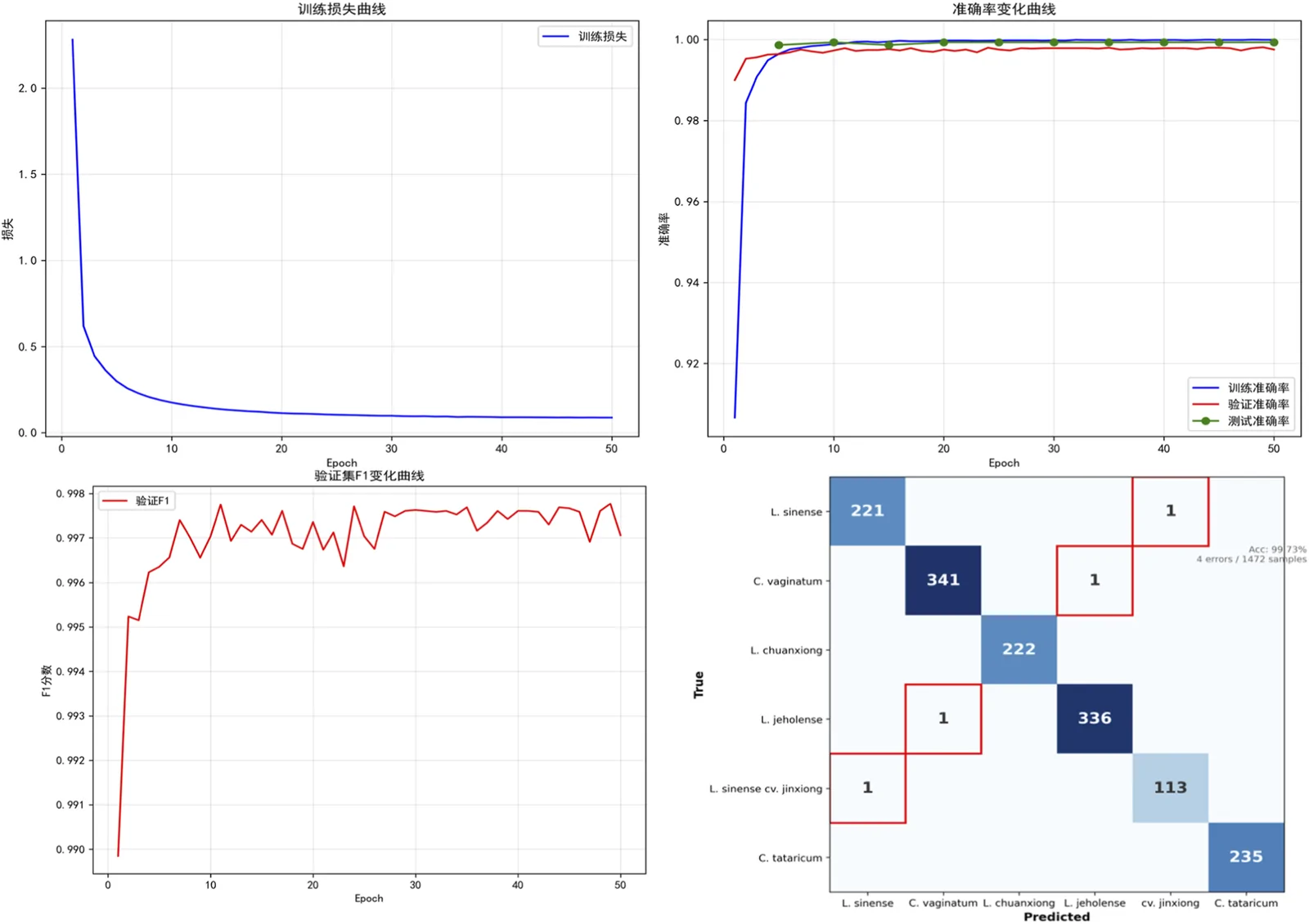
Confusion matrix of multi-teacher distilled MobileNetV2 for Ligustici Rhizoma et Radix varieties.

Although MobileNetV2 offers the advantages of a small parameter count and high inference speed, its limited feature extraction capability restricts the accurate capture of discriminative characteristics among *Ligustici Rhizoma et Radix* and its adulterants. Consequently, its key performance metrics, including accuracy and precision, were insufficient for practical identification. To overcome this limitation, knowledge distillation was introduced in this study.

The experimental results showed that single-teacher distillation simultaneously improved accuracy, precision, recall, and F1-score while maintaining an essentially unchanged model parameter count and inference efficiency, preliminarily demonstrating the feasibility of transferring knowledge from a teacher model to a lightweight student model. Among the single-teacher configurations, ResNet50 provided the most effective supervision, achieving an accuracy of 99.32%, compared with 96.20% for VGG16% and 97.21% for DenseNet169. This result suggests that the deep feature representations learned by the residual network were more compatible with the lightweight architecture of the student model.

During dual-teacher distillation, none of the teacher combinations surpassed the accuracy achieved using ResNet50 alone. Instead, their performance was inferior to that of the ResNet50 single-teacher configuration. This finding suggests that differences in feature distributions among heterogeneous backbone networks may have introduced representational conflicts or mutual interference during dual-teacher fusion, thereby reducing the effectiveness of knowledge transfer.

The three-teacher collaborative distillation strategy ultimately achieved the best overall performance, with accuracy, precision, recall, and F1-score values of 99.73%, 99.71%, 99.70%, and 99.70%, respectively. These values exceeded those obtained with all single-teacher and dual-teacher configurations. The results indicate that the three-teacher framework facilitated complementary knowledge integration from the heterogeneous VGG16, DenseNet169, and ResNet50 backbones and mitigated the representational conflicts observed in the dual-teacher configurations.

The confusion matrix (Figure 13) shows four misclassifications clustered within two taxonomically relevant species pairs: reciprocal misidentifications between C. vaginatum and L. jeholense (two errors), as well as between L. sinense and L. sinense cv. Jinxiong (another two errors). By contrast, L. chuanxiong and C. tataricum were classified with zero misidentifications.

The misclassification between C. vaginatum and L. jeholense is probably attributable to processing-mediated morphological convergence. Following commercial slicing and drying, the conical rhizome of C. vaginatum and the strip-shaped rhizome of L. jeholense may exhibit superficial resemblance. Consistent with the taxonomic revisions of this complex based on fruit morphology (Pimenov et al., 2001), vegetative characters alone are often insufficient for reliable discrimination. Meanwhile, the bidirectional confusion between L. sinense and its cultivar jinxiong arises from their intrinsic biological affinity: both possess block-shaped rhizomes with dark brown to black outer surfaces, differing only in subtle cross-sectional coloration. Consequently, differentiation remains challenging even for experienced specialists (Zheng et al., 2024).

Overall, the three-teacher distilled model retained the lightweight advantages of MobileNetV2, including 2.23 million parameters, an inference speed of 294 FPS, and a computational cost of 0.33 GFLOPs, while compensating for the limited feature representation capability of the original model through multi-source knowledge aggregation. It therefore achieved a favorable balance between identification accuracy and computational efficiency.

### 5.3 K-fold cross-validation

K-fold cross-validation is a commonly used model evaluation strategy in machine learning. In this approach, the dataset is divided into (k) subsets. In each iteration, (k-1) subsets are used for model training, while the remaining subset is used for evaluation. This procedure is repeated (k) times, with each subset serving once as the held-out fold. By reducing the influence of a single data partition, K-fold cross-validation provides a more comprehensive assessment of model performance and improves the stability and reliability of the evaluation results (Hu and Zhu, 2025).

Five-fold cross-validation ((K = 5)) was employed in this study,The cross-validation performance of the original MobileNetV2 model and the three-teacher distilled model is compared in Figure 14.

**FIGURE 14:**
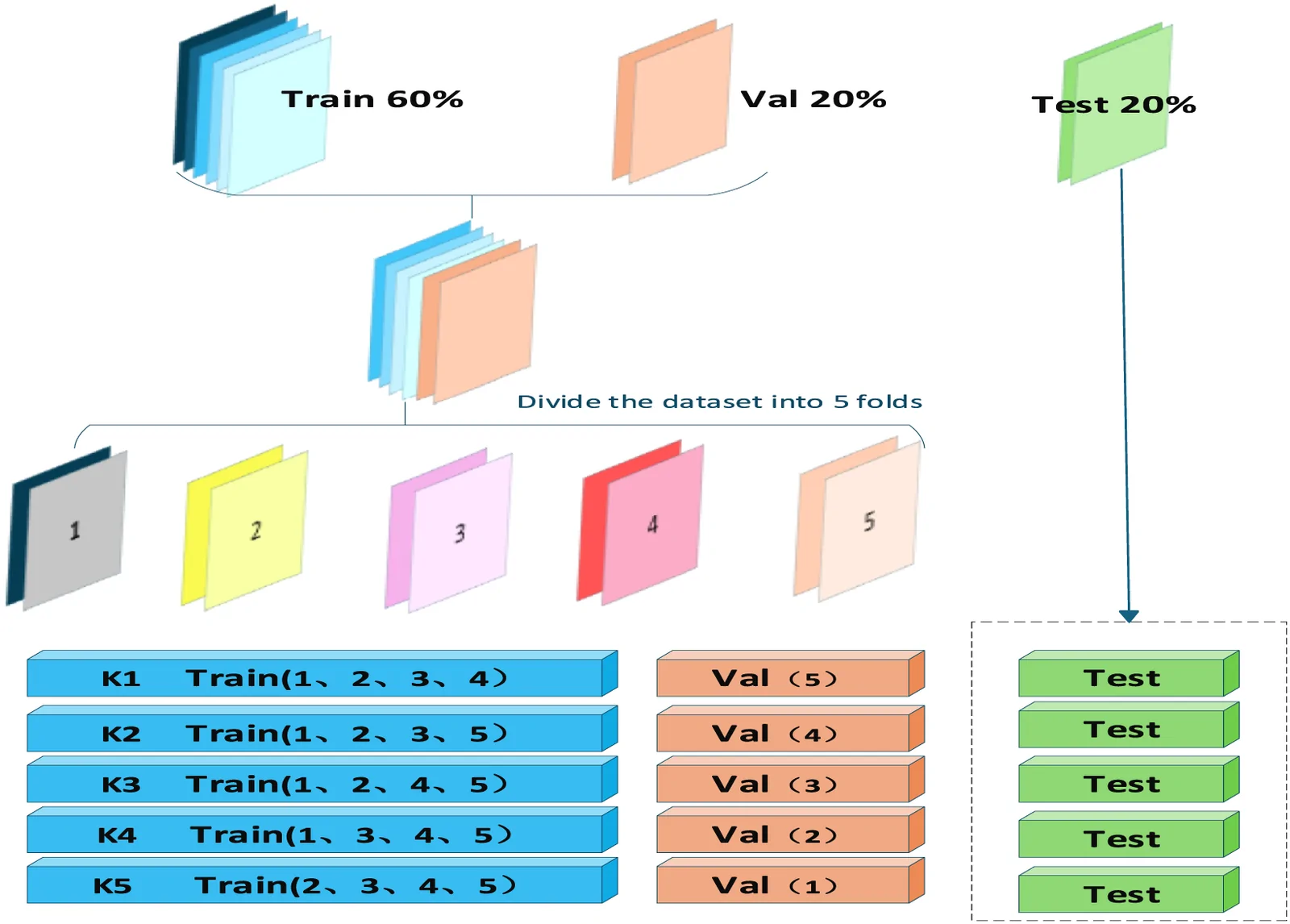
Data allocation in K-fold cross-validation.

### 5.4 Model validation

To comprehensively evaluate the reliability of the model, five-fold cross-validation ((K = 5)) and gradient-weighted class activation mapping (Grad-CAM) were employed. Grad-CAM heatmaps were used to determine whether the discriminative features extracted by the model were concentrated on the decoction pieces themselves rather than on irrelevant background regions, thereby evaluating the reliability and interpretability of the identification results (Huang et al., 2018; Hu and Zhu, 2025).

#### 5.4.1 Five-fold cross-validation

The combined training and validation data were evenly divided into five subsets. In each round, four subsets were used for model training, while the remaining subset was used for validation. This procedure was repeated for five rounds, with each subset serving once as the validation set. The classification accuracy, generalization ability, and stability of the model were comprehensively evaluated across the five folds. This strategy reduced the evaluation bias associated with a single dataset partition and improved the reliability of the results. The cross-validation procedure is illustrated in Figure 15, and the corresponding results are presented in Table 7 and Figure 15.

**FIGURE 15:**
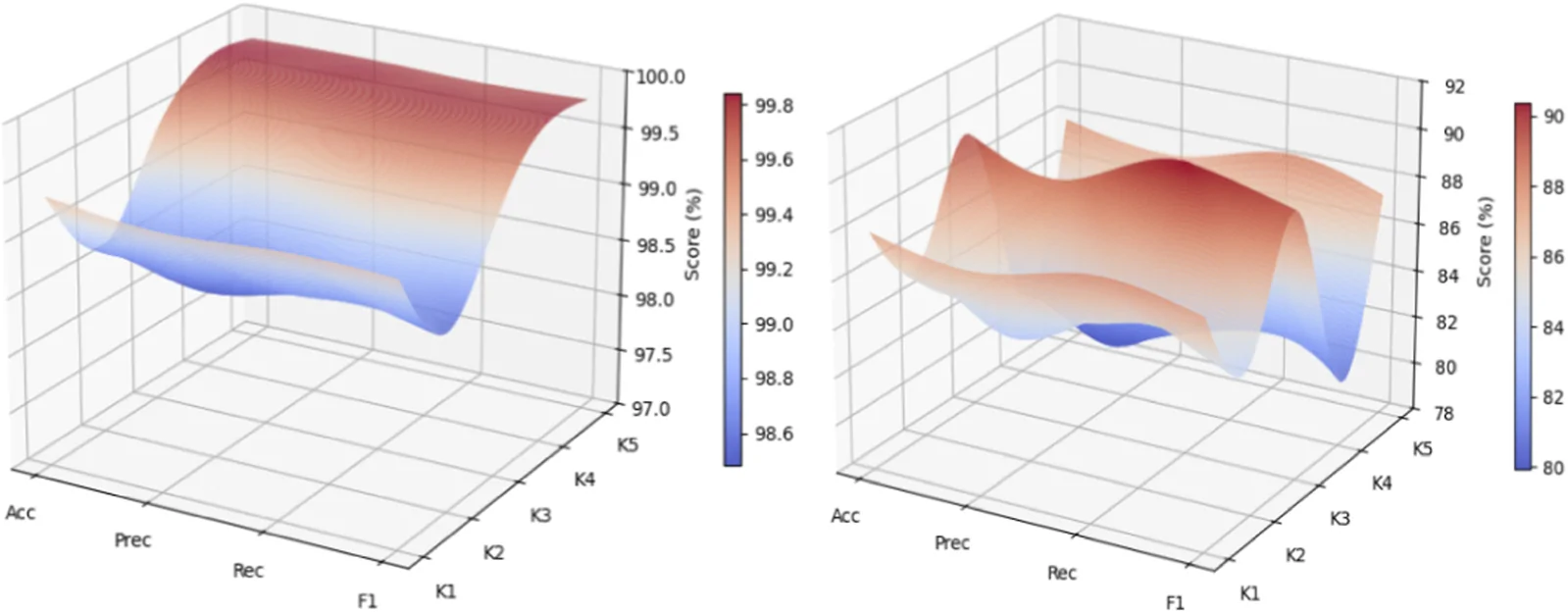
Quantitative metrics and 3D surface visualization of model performance over five cross-validation folds.

**TABLE 7:** 5-Fold cross-validation under two network models.

Model	Performance evaluation				Model parameters		Inference efficiency	
MobileNetV2	Acc	Prec	Rec	F1	M	Size	FPS	FLOPs
K1	87.89%	87.23%	88.13%	87.45%	2.23	8.75	302	0.33
K2	85.61%	83.62%	85.63%	84.36%	2.23	8.75	273	0.33
K3	89.52%	88.56%	90.14%	89.10%	2.23	8.75	289	0.33
K4	81.42%	80.24%	81.95%	80.79%	2.23	8.75	276	0.33
K5	87.92%	87.26%	88.27%	87.36%	2.23	8.75	291	0.33
Mean	86.47%	85.38%	86.82%	85.78%	2.23	8.75	286.2	0.33

## 6 Results analysis

In the five-fold cross-validation, the distilled model exhibited only minimal variation across folds, with the accuracy remaining stable within the range of 99.40% ± 0.47%. The inter-fold fluctuation was substantially smaller than that of MobileNetV2, which showed an accuracy of 86.47% ± 3.15%. These results indicate that knowledge distillation effectively improved the adaptability of the model to variations in data partitioning and enhanced its generalization performance.

In terms of computational characteristics, the distilled model had the same number of parameters, model size, and FLOPs as the student model MobileNetV2, while its inference frame rate was also essentially the same as that of MobileNetV2.

Thus, the distilled model inherited the high classification accuracy of the teacher models while retaining the small parameter count and fast inference speed of the lightweight student model. This ensures the deployment advantages of a lightweight architecture and makes the model suitable for resource-constrained devices, including smartphones, tablets, and other embedded mobile platforms.

### 6.1 Grad-CAM heatmap visualization analysis

#### 6.1.1 Grad-CAM heatmap visualization

Grad-CAM was used to convert the latent feature extraction process of the model into visual heatmaps, thereby revealing whether the model focused on the core discriminative regions of the medicinal materials during identification (Huang et al., 2018; Hu and Zhu, 2025).

To verify the reliability of the fusion model in decision-making, Grad-CAM heatmaps were used to compare the regions of interest among models (Figure 16). In each row, the images are arranged from left to right as follows: the original sample, the heatmap of the large-scale model (teacher model), and the heatmap of the distilled model. The results showed that the high-response regions of the distilled model heatmaps, represented by red and yellow areas, largely overlapped with those of the teacher model and were concentrated on key discriminative features of the decoction pieces, such as texture and morphological edges. This indicates that the distilled model inherited the core feature-capturing capability of the teacher model. Compared with the original MobileNetV2 model, the fused model further reduced attention to redundant regions through knowledge transfer, resulting in heatmaps that were more focused on effective discriminative features while preserving the key feature discrimination capability of the teacher model.

**FIGURE 16:**
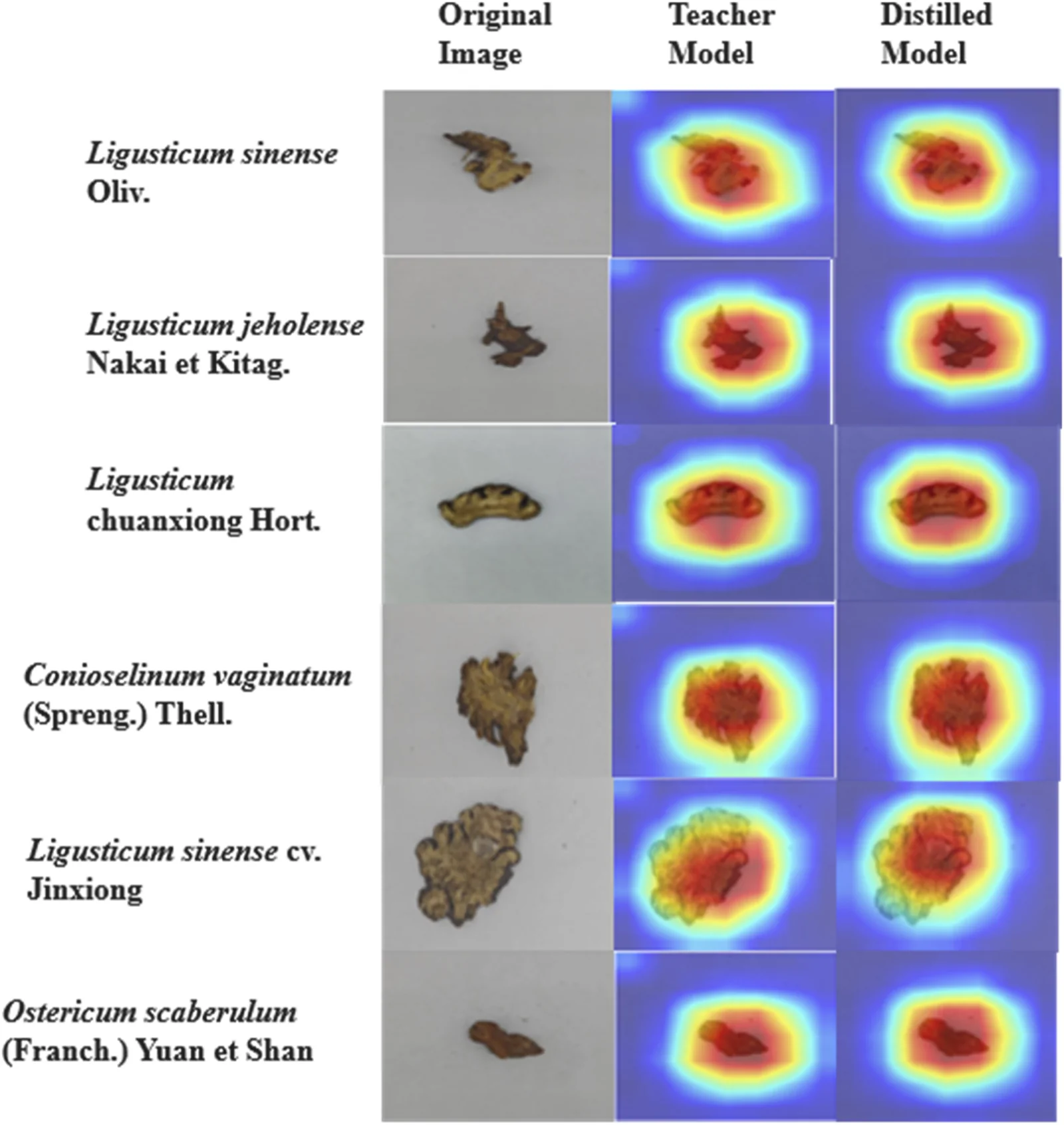
Analysis of botanical drug Piece Heatmap.

### 6.2 Development of a PyQt5-Based Image identification application for decoction pieces

An image identification application for traditional Chinese medicine decoction pieces was developed using PyQt5 and the distilled model. A modular design was adopted to integrate the complete workflow, including model loading, image preprocessing, inference and prediction, and result analysis. A graphical user interface was developed to enable interactive operation of the identification system. Figure 17.

**FIGURE 17:**
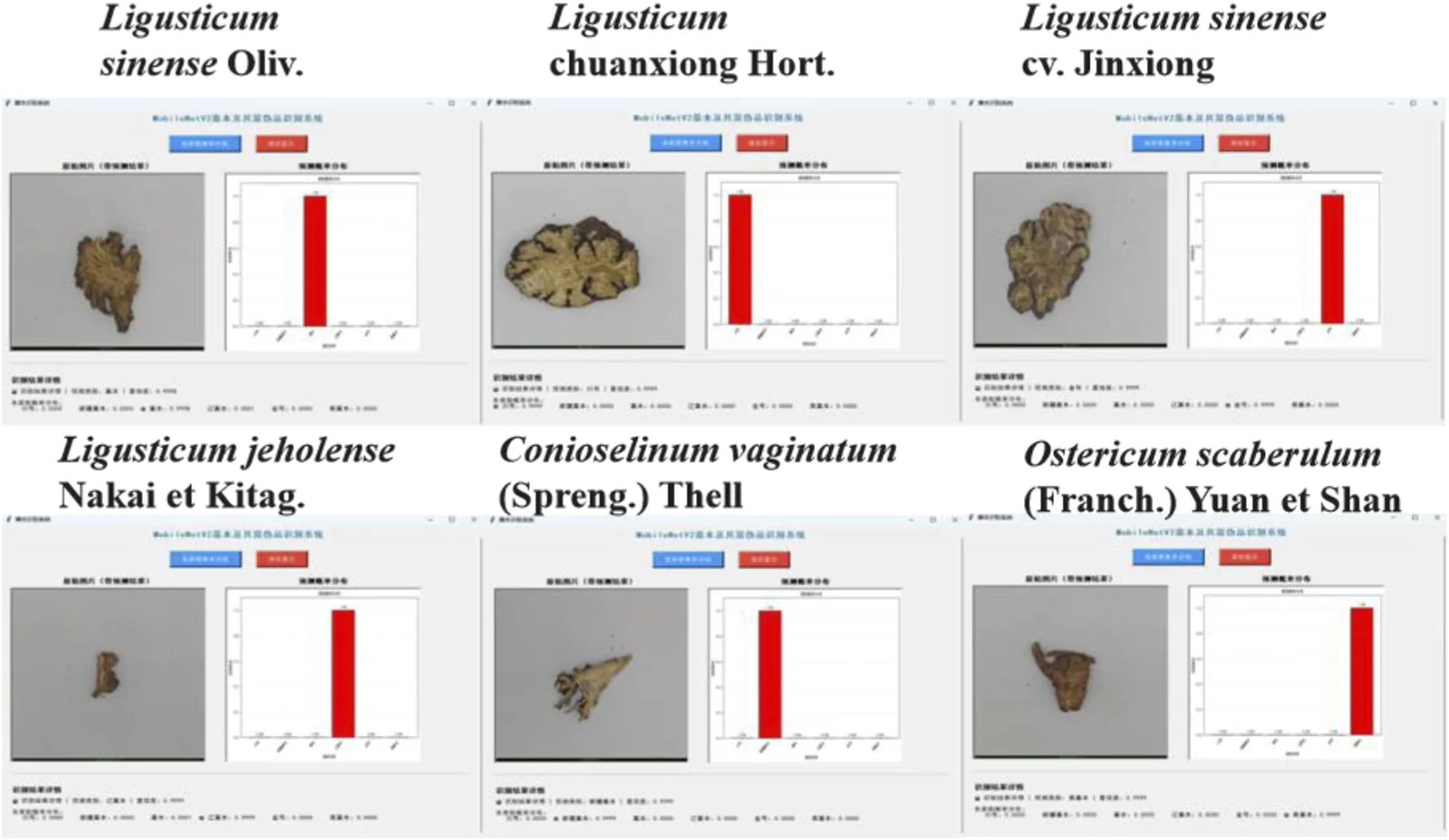
PyQt5-Based botanical drugl Piece Image Recognition.

## 7 Discussion

Traditional Chinese medicines constitute an important material basis for disease prevention and treatment in traditional Chinese medicine. The quality of medicinal materials is directly related to patient health and medication safety and is also essential for ensuring clinical efficacy. The fundamental determinants of the quality of traditional Chinese medicine decoction pieces include the authenticity and overall quality of their source materials. China has a long history of traditional Chinese medicine use, involving numerous medicinal species and geographically diverse production areas. Differences in historical records, regional terminology, and medication practices, together with the continuous emergence of analogous and substitute materials, have resulted in widespread phenomena such as different medicinal materials sharing the same name and the same medicinal material being known by different names. Multiple botanical origins are also common for a single medicinal material. Furthermore, the highly similar morphological characteristics of medicinal materials from closely related taxa and the regional commingling of officially recognized materials with traditionally used substitutes present considerable challenges to accurate species identification, 509 thereby potentially affecting the efficacy and safety of clinical medication.

In recent years, deep learning has emerged as a promising technique for the intelligent identification of traditional Chinese medicines owing to its powerful feature extraction capability; however, balancing identification accuracy and deployment efficiency remains a critical challenge. Targeting this issue, the present study selected Ligustici Rhizoma et Radix decoction pieces and their common adulterants as the research objects and constructed a heterogeneous multi-teacher knowledge distillation strategy using three high-performance models (ResNet50, DenseNet169, and VGG16) as teachers. By incorporating a dual-loss fusion mechanism and temperature scaling strategy, the discriminative knowledge embedded in these large teacher models was effectively transferred to the lightweight MobileNetV2 student model, finally yielding a lightweight, deployable, and high-accuracy intelligent identification model for medicinal materials. Consistent with the dual-teacher distillation scheme recently proposed by Hou et al. (Zeiler et al., 2014), the three-teacher distillation paradigm developed in this study further demonstrates the broad applicability of knowledge distillation as an effective model compression and performance enhancement strategy for botanical drug image recognition.

This study used image-based feature information to address the practical need for rapid on-site identification during the circulation of traditional Chinese medicines. The resulting lightweight intelligent model is potentially suitable for portable devices such as smartphones, tablets, and embedded terminals and may therefore support applications in resource-constrained settings, 524 including retail pharmacies, medicinal-material markets, procurement inspections, and sampling inspections. Although the distilled model achieved a favorable balance between high accuracy and lightweight deployment in identifying Ligustici Rhizoma et Radix decoction pieces and their adulterants, several limitations remain. First, the study focused exclusively on Ligustici Rhizoma et Radix and its adulterants and did not include other readily confused traditional Chinese medicinal materials. Nevertheless, the proposed lightweight identification framework based on heterogeneous multi-teacher knowledge distillation provides a potentially transferable approach for traditional Chinese medicine quality control and may contribute to the modernization and international development of traditional Chinese medicine. Second, the image acquisition conditions and the diversity of the included samples were relatively limited and did not fully represent the complex environments encountered during actual market circulation. Therefore, the adaptability and robustness of the model in real-world application scenarios require further improvement and valida.

## Data Availability

The original contributions presented in the study are included in the article/supplementary material, further inquiries can be directed to the corresponding author/s.
